# Individualizing Injectable Testosterone Replacement Therapy in Primary Care: Pharmacokinetics, Symptom Stability, Safety Monitoring, and Injection Frequency

**DOI:** 10.7759/cureus.110570

**Published:** 2026-06-10

**Authors:** Khashayar Farzam

**Affiliations:** 1 Health Sciences, McMaster University, Kitchener, CAN; 2 Metabolic Science, True North Metabolic, Kitchener, CAN

**Keywords:** endocrinology, hypogonadism, low testosterone, men's health, testosterone deficiency, testosterone replacement therapy (trt)

## Abstract

Injectable testosterone is a familiar treatment option for men with confirmed hypogonadism, but the interval between injections is sometimes chosen out of habit rather than clinical reasoning. Short-acting testosterone esters such as testosterone cypionate and enanthate can produce meaningful changes in serum exposure across the dosing cycle, particularly when larger doses are given at longer intervals. For some patients, this may create a pattern of early peak-related symptoms and late-cycle recurrence of hypogonadal symptoms. It can also make laboratory interpretation and safety monitoring more difficult. This article reviews a practical approach to injectable testosterone therapy in primary care. Injection frequency should only be considered after the diagnosis of hypogonadism has been properly established, including compatible symptoms, repeat morning testosterone testing, and appropriate interpretation of total testosterone, free or calculated free testosterone, sex hormone-binding globulin (SHBG), and gonadotropins. Once treatment is indicated, clinicians should document the testosterone ester, dose, concentration, route, injection interval, timing of the last injection, and timing of bloodwork. Without that context, testosterone results can be misleading. Every-two-week dosing remains convenient, but it should not be the automatic default for short-acting injectable testosterone. Weekly dosing is often a reasonable and effective schedule, and many patients do well with it. However, some men continue to report symptom cycling, early acne or oily skin, mood or sleep disturbance, breast tenderness, fluid retention, late-cycle fatigue, reduced libido, or erectile symptoms. In these cases, the issue may be dose timing and peak-trough variation rather than inadequate total weekly dose. More frequent lower-dosage regimens, such as twice-weekly, three-times-weekly, every-other-day, or daily injections, should be viewed as optional tools rather than universal standards. They may be useful when symptoms, hematocrit trends, estradiol-related symptoms, blood pressure changes, fluid retention, low SHBG, or patient preference suggest that a smoother exposure pattern would be helpful, in conjunction with a dose adjustment. Conversely, if weekly dosing provides stable symptoms and acceptable monitoring results, additional injection frequency may add burden without clear benefit. The goal of injectable testosterone therapy is not to maximize testosterone levels. It is to achieve physiologic replacement with stable symptoms, interpretable laboratory results, tolerable treatment burden, and appropriate safety monitoring. In many patients, dose redistribution should be considered before dose escalation. This is a practical review for primary care clinicians with the goal of having an additional clinical tool for managing testosterone replacement therapy dosing.

## Introduction and background

Testosterone therapy should be used for men with symptoms and signs of testosterone deficiency and consistently low testosterone concentrations, not as a general treatment for fatigue, aging, body composition concerns, or performance enhancement [1-4]. This distinction matters because injectable testosterone is often discussed as though the main clinical decision is simply whether to prescribe it. In practice, the diagnosis is only the first step. Once treatment is indicated and an injectable formulation is chosen, the dose, route, interval, monitoring plan, and interpretation of follow-up laboratory results all become part of the therapeutic decision [1-6].

Primary care clinicians frequently encounter men who are already using injectable testosterone, men who have been started elsewhere, or men who are transitioning from another formulation because of cost, absorption, preference, or access. In these situations, clinicians may inherit older prescribing habits rather than deliberately design a regimen around the patient’s symptoms, risk factors, and laboratory pattern. Testosterone cypionate or enanthate given every two weeks remains familiar, easy to schedule, and consistent with some product labeling, but convenience should not be mistaken for physiologic replacement [5-8]. A regimen that is simple to prescribe may still be difficult to interpret clinically if symptoms and bloodwork vary across the dosing cycle [5-8].

The underlying problem is pharmacokinetic in nature. Larger injections given at longer intervals produce higher early peaks in serum testosterone and lower late troughs [5-8]. That pattern may matter for symptoms, laboratory interpretation, hematocrit, estradiol, blood pressure, fluid retention, and patient satisfaction [1-8]. It can also create a common clinical trap: a patient may appear overtreated shortly after an injection and undertreated near the end of the interval. Without knowing when the bloodwork was drawn relative to the last injection, a testosterone result can be misinterpreted, and the response may be dose escalation when the more logical adjustment is dose redistribution [1-8].

Weekly dosing is often a reasonable and effective improvement, and many patients do well on it. For that reason, this article does not argue that every patient requires highly frequent injections. A stable patient with good symptom control, interpretable laboratory results, acceptable hematocrit, and no meaningful peak-related adverse effects may not benefit from added complexity [1-6]. However, weekly dosing should not be treated as the endpoint of individualization. Some patients remain sensitive to fluctuations even with weekly injections, especially when symptoms repeatedly track the injection cycle or when laboratory and safety markers suggest that peak exposure may be clinically relevant [5-8].

This issue is particularly important because testosterone therapy is not monitored by the testosterone value alone. The clinical picture includes symptom timing, adverse effects, hematocrit, blood pressure, estradiol-related symptoms, fertility goals, cardiometabolic risk, and patient adherence [1-4]. A more frequent lower-dose schedule may reduce dose per injection and smooth exposure for selected patients, but it also increases treatment burden [5-8]. The goal is therefore not to make injection schedules as frequent as possible. The goal is to find the least burdensome regimen that provides stable symptom control, physiologic replacement, interpretable monitoring, and an acceptable safety profile [1-8].

This article proposes a practical approach for primary care: avoid using every-two-week injections as an unexamined default choice, recognize that weekly dosing works for many patients, and consider more frequent lower-dose schedules when cyclic symptoms or safety signals suggest avoidable peak-trough variation. Dose redistribution should generally be considered before total dose escalation when the clinical pattern suggests fluctuation rather than under-replacement [1-8].

This review aims to provide a practical framework for primary care clinicians to individualize short-acting injectable testosterone dosing intervals, symptom monitoring, and safety surveillance.

## Review

Diagnosis before injection optimization

Injection frequency should only be discussed after the indication for testosterone therapy is confirmed. Hypogonadism is a clinical and biochemical diagnosis. Typical symptoms include low libido, reduced morning erections, erectile dysfunction, fatigue, low mood, reduced muscle mass or strength, reduced exercise tolerance, increased fat mass, and, in selected cases, anemia or osteoporosis [1-4]. Symptoms are nonspecific, so biochemical confirmation is essential.

Testing should include morning total testosterone, repeated when low or borderline. Free testosterone is also essential, especially when the total testosterone level is borderline or sex hormone-binding globulin (SHBG) is abnormal [1-4]. SHBG and albumin are needed for calculating free testosterone. Luteinizing hormone (LH) and follicle-stimulating hormone (FSH) are gonadotropins and help distinguish primary from secondary hypogonadism and compose an essential part of the assessment of hypogonadism. Prolactin, pituitary assessment, thyroid testing, iron studies, sleep apnea assessment, medication review, and evaluation for obesity or systemic illness may be appropriate depending on the clinical picture [1-4].

Before treatment, clinicians should document hematocrit, blood pressure, cardiometabolic risk, fertility goals, and prostate assessment where age and risk make this relevant [1-4]. Optimizing injection frequency cannot compensate for an uncertain diagnosis. In addition, the treatment of hypogonadism does not routinely fix cardiometabolic risk factors.

Laboratory units and timing

Testosterone literature is complicated by inconsistent units. Canada, the United Kingdom, Australia, Hong Kong, and many other jurisdictions commonly report total testosterone in nmol/L. United States publications often use ng/dL. Total testosterone can be converted approximately by multiplying nmol/L by 28.8. Thus, 10.4 nmol/L is approximately 300 ng/dL. Estradiol may be reported in pmol/L or pg/mL, with 1 pg/mL approximately equal to 3.67 pmol/L. Hematocrit may be reported as L/L or percentage, so 0.54 L/L equals 54%.

Timing also matters as much as units. A testosterone level drawn 24-48 hours after a large injection does not mean the same thing as a trough level drawn before the next dose. Every-two-week dosing makes this problem especially prominent: the same patient can appear overtreated early and undertreated late. A requisition or clinic note should document the ester, dose, concentration, route, injection interval, date and time of last injection, and date and time of blood draw. A testosterone result without injection timing is often an incomplete result, as it lacks clinical context.

Pharmacokinetic limitations of legacy injection schedules

Testosterone cypionate and enanthate are esterified depot preparations of testosterone. After an injection, ester hydrolysis releases testosterone over time, producing a rise, peak, and subsequent decline [5-8]. A pharmacokinetic study of 200 mg intramuscular testosterone cypionate in hypogonadal men showed marked fluctuation over two weeks, with supraphysiologic mean peak concentrations around days 4 to 5 and substantially lower concentrations by day 14 [7]. Studies comparing transdermal preparations with intermittent injections have also shown that short-acting intramuscular esters can produce wider fluctuations than steadier daily formulations [8].

The adverse effects can be most prominent with every-two-week dosing because it maximizes the fluctuation among common short-acting ester regimens. Patients may feel overstimulated, irritable, or fluid-retentive or have nipple tenderness shortly after their injection and then be fatigued, have low libido, or be symptomatic before the next injection. If only trough levels are checked, clinicians may increase the total dose when the more logical response is shortening the interval and lowering the dose per injection.

Weekly dosing reduces this instability and is often a sensible, practical schedule for some patients. It is easier to follow than very frequent dosing and works well for many men. But weekly dosing is not inherently optimal for every patient and can suffer from some of the same risks that biweekly dosing does. Some patients still report early peak effects or late-cycle symptoms, especially with larger weekly doses, low SHBG, high symptom sensitivity, or fluctuating estradiol symptoms. More frequent lower-dose regimens reduce dose-per-injection and may smoothen serum exposure, although direct comparative outcome trials remain limited.

Traditional versus more frequent lower-dose regimens

The real comparison is not weekly versus twice weekly. It is a larger intermittent exposure versus an individualized lower-dose exposure. Every-two-week dosing offers fewer injections and may be acceptable for selected patients who feel stable and have safe monitoring. It should not, however, be the unexamined default choice for short-acting ester testosterone injections. Weekly dosing is a more defensible common compromise. It reduces peak-trough amplitude, simplifies routine care, and may be sufficient for many patients.

More frequent lower-dose dosing includes twice weekly, three times weekly, every other day, or daily administration. These schedules lower the amount of testosterone delivered at one time. They may be useful when symptoms repeatedly track the injection cycle, when estradiol-related symptoms appear early after dosing, when hematocrit rises with high measured levels, when blood pressure or fluid retention worsens after injections, or when low SHBG complicates the interpretation of laboratory test findings. The downside is the logistical issue with more frequent injections. More injections can reduce adherence, increase anxiety around treatment, and create unnecessary complexity if weekly dosing is already effective. Hence, treatment plans need to be customized to the patient's needs.

The clinical aim is therefore not to prescribe the most frequent regimen. It is to find the least burdensome schedule that maintains stable symptoms, physiologic testosterone exposure, and acceptable safety markers.

Mechanistic considerations: why peak exposure may matter

Testosterone and dihydrotestosterone act through the androgen receptors in multiple tissues. Higher peaks may amplify androgenic effects in susceptible skin, hair follicles, prostate tissue, mood regulation, and the erythropoietic system. Acne, oily skin, androgenic alopecia progression, irritability, or sleep disruption shortly after injection should prompt consideration of the dose size and peak exposure rather than automatic escalation or add-on therapy [9].

Aromatization is also relevant. Testosterone is converted to estradiol by the aromatase enzyme. Estradiol is not merely an adverse metabolite. It is important for libido, sexual function, body composition, cardiac health, and bone health in men [10-13]. Large testosterone peaks increase the substrate availability for aromatization and may contribute to breast tenderness, fluid retention, or mood changes in susceptible patients. This does not mean estradiol should be suppressed routinely. Aromatase inhibition can lower estradiol, but over-suppression may worsen libido, erectile function, joints, mood, and bone density, alongside unknown side effects. This provides another example of where dosage adjustment may address the underlying issue rather than adjunctive therapy such as aromatase inhibitors [12,13].

Testosterone also stimulates erythropoiesis. Human studies support the role of testosterone for erythropoietin stimulation, hepcidin suppression, and increased iron availability [14,15]. Injectable testosterone has been associated with higher erythrocytosis rates than some transdermal preparations [16-19]. It is biologically plausible that higher peaks of testosterone may contribute to the erythropoietic drive, although direct trials comparing injection frequencies for hematocrit outcomes are limited.

Blood pressure and fluid balance deserve similar attention. Androgens can influence renal sodium and water handling, plasma volume, vascular tone, and the renin-angiotensin-aldosterone system pathways [20-22]. The US Food and Drug Administration recently required class-wide testosterone labeling changes related to blood pressure after ambulatory blood pressure studies [23]. Lipid effects are also variable. HDL suppression is most pronounced with oral anabolic steroids and supraphysiologic androgen exposure, but testosterone therapy can still influence lipids depending on formulation, dose, achieved levels, and population studied [24,25].

These mechanisms do not prove that frequent injections improve long-term cardiovascular outcomes. They do support avoiding unnecessarily high peaks when stable physiologic replacement can be achieved with lower per-injection doses.

Symptom stability and safety monitoring

Patients should be asked how they feel in the first 24-72 hours after injection and before the next dose. Early-cycle symptoms may include acne, oily skin, insomnia, irritability, breast tenderness, edema, headache, or blood pressure symptoms. Late-cycle symptoms may include fatigue, low libido, erectile symptoms, low mood, brain fog, reduced morning erections, or poor exercise recovery.

Safety monitoring should extend beyond the testosterone serum level. Hematocrit should be checked at baseline and after initiation of TRT or dose changes, then periodically according to guideline-based care [1-4]. A rising hematocrit should prompt a review of achieved testosterone levels, injection interval, dose-per-injection, sleep apnea, smoking, hypoxia, dehydration, and cardiopulmonary disease. Phlebotomy may be rarely needed, but repeated phlebotomy should not become a substitute for correcting the treatment protocol.

Estradiol should also be interpreted clinically and within the context of the treatment protocol. An elevated estradiol value without symptoms should not automatically lead to an aromatase inhibitor. If breast tenderness, fluid retention, or mood symptoms appear after injections, clinicians should first review total testosterone dose, peak timing, adiposity, alcohol intake, sleep apnea, and whether the same weekly amount could be delivered in smaller, more frequent doses. It is worth noting that validated data on serum estradiol laboratory ranges remain limited in men. In addition, the use of aromatase inhibitors lacks long-term safety data in men as well.

Blood pressure should be measured before and after starting therapy and after meaningful dose or frequency changes. Lipids should be followed in the broader context of cardiovascular risk, particularly in men with obesity, diabetes, hypertension, smoking history, or established cardiovascular disease. Fertility must be discussed before therapy because exogenous testosterone suppresses LH and FSH, reduces intratesticular testosterone, and can impair spermatogenesis [26-29]. Dividing the dose does not preserve fertility, and patients should be counseled on this.

Individual factors affecting regimen choice

SHBG is central to the interpretation of testosterone testing. Low SHBG is common with obesity, insulin resistance, metabolic syndrome, hypothyroidism, glucocorticoid exposure, and androgen exposure. It may make total testosterone appear low while free testosterone is less reduced. High SHBG can make total testosterone appear acceptable while free testosterone is low. SHBG is not an injection-frequency calculator, but it is essential for deciding whether the total testosterone reflects biologically available androgen exposure [1-4,30].

Some clinicians find that low-SHBG patients report more symptom fluctuation and may tolerate smaller, more frequent doses better, but this should be presented as a clinical observation rather than settled evidence. Adiposity also matters because it can lower SHBG, increase aromatization, worsen sleep apnea risk, and increase cardiometabolic risk. Intramuscular and subcutaneous routes may both be considered. Smaller volumes can make more frequent subcutaneous administration better tolerated for selected patients, and published data support subcutaneous testosterone as a feasible option in some settings [31-33].

Adherence remains the limiting factor. A theoretically smoother regimen is not better if the patient cannot follow it.

Interpreting transdermal safety data in injectable practice

The TRAVERSE trial provided important reassurance that testosterone gel, used in appropriately selected middle-aged and older men with hypogonadism and high cardiovascular risk, did not increase major adverse cardiovascular events compared with placebo [34]. That evidence is clinically important, but it should not be overextended. Transdermal testosterone provides steadier daily exposure than large intermittent injections. A trial using topical testosterone cannot fully answer whether every-two-week injectable regimens create avoidable peak-related symptoms or monitoring problems.

For practical reference, common testosterone-related units and interpretation issues, injectable testosterone dosing patterns, and mechanisms relevant to monitoring are summarized in Tables 1-3.

**Table 1:** Interpretation of common testosterone-related units across jurisdictions SHBG = sex hormone-binding globulin

Analyte	Common Canadian/international unit	Common US unit	Approximate conversion	Practical note	Supporting references
Total testosterone	nmol/L	ng/dL	1 nmol/L ≈ 28.8 ng/dL	Always report the unit with any threshold. Example: 10.4 nmol/L ≈ 300 ng/dL.	[1-4]
Free testosterone	pmol/L	pg/mL or ng/dL	Method dependent	Calculated values vary by equation, assay inputs, SHBG, and albumin. Avoid comparing across laboratories without a method context.	[1-4,30]
Estradiol	pmol/L	pg/mL	1 pg/mL ≈ 3.67 pmol/L	Sensitive assays are preferred when available in men. Interpret with symptoms and testosterone timing.	[10-13]
Hematocrit	L/L	%	0.54 L/L = 54%	Guideline thresholds vary slightly by clinical context. Rising hematocrit should prompt reassessment of dose, interval, and comorbid contributors.	[1-4,14-19]
Testosterone dose	mg/week or mg/injection	mg/week or mg/injection	Same	Specify ester, concentration, route, dose per injection, total weekly dose, and interval.	[1-8,31-33]

**Table 2:** Mechanisms and monitoring domains affected by testosterone exposure AI = aromatase inhibitor; apoB = apolipoprotein B; CBC = complete blood count; DHT = dihydrotestosterone; hCG = human chorionic gonadotropin; HDL = high-density lipoprotein; LDL = low-density lipoprotein; LH/FSH = luteinizing hormone/follicle-stimulating hormone; RAAS = renin-angiotensin-aldosterone system; SERM = selective estrogen receptor modulator; SHBG = sex hormone-binding globulin; TRT = testosterone replacement therapy

Domain	Basic mechanism	Clinical signal	Monitoring or practical response	Supporting references
Erythropoiesis	Erythropoietin stimulation, hepcidin suppression, and increased iron availability.	Rising hematocrit or hemoglobin.	Monitor CBC. Assess achieved levels, dose, interval, sleep apnea, smoking, hypoxia, dehydration, and cardiopulmonary disease. Consider dose reduction, interval adjustment, formulation change, or temporary hold when indicated.	[1-4,14-19]
Estradiol	Aromatization of testosterone to estradiol, with higher testosterone exposure increasing substrate availability.	Breast tenderness, edema, mood or libido change, or fluid retention in selected patients.	Treat symptoms and context, not the estradiol number alone. Review dose, timing, adiposity, alcohol, and sleep apnea before aromatase inhibitor use.	[10-13]
Blood pressure and fluid balance	Possible effects on sodium and water handling, plasma volume, vascular tone, and RAAS-related pathways.	Rising blood pressure, edema, headache, or weight fluctuation.	Monitor blood pressure before and after initiation or dose changes. Review fluid retention, hematocrit, sleep apnea, NSAIDs, stimulants, sodium, alcohol, and cardiovascular risk.	[20-23]
Lipids	Androgen effects on hepatic lipid metabolism; HDL suppression is more prominent with oral anabolic steroids and supraphysiologic exposure.	HDL reduction or worsening non-HDL cholesterol, LDL cholesterol, or apoB context.	Monitor lipids and global cardiovascular risk. Avoid high-normal or supraphysiologic exposure when physiologic replacement is the goal.	[1-4,24,25,34]
Fertility	LH/FSH suppression, reduced intratesticular testosterone, and impaired spermatogenesis.	Oligospermia, azoospermia, testicular atrophy, or infertility concerns.	Counsel before TRT. Consider semen analysis, fertility preservation, hCG/SERM-based approaches, or specialist referral when fertility is desired.	[1-4,26-29]
Skin, hair, androgen-sensitive tissues	Androgen receptor and DHT exposure in susceptible tissues.	Acne, oily skin, androgenic alopecia progression, or relevant lower urinary tract symptom context.	Review dose size and peak timing. Discuss patient priorities and manage dermatologic or urologic concerns when clinically relevant.	[1-4,9]

**Table 3:** Legacy versus individualized injectable testosterone schedules

Regimen category	Example interval	Expected pharmacokinetic pattern	Advantages	Limitations	Clinical role	Supporting references
Legacy longer-interval dosing	Every two weeks	Largest peak-trough variation among common short-acting ester schedules.	Fewer injections; simple schedule; familiar to clinicians.	Early peaks, late troughs, symptom cycling, and lab timing problems.	May work for selected stable patients, but should not be an unexamined default.	[5-8]
Weekly dosing	Every seven days	Less fluctuation than every-two-week dosing, but still intermittent.	Practical, familiar, often effective; lower burden than more frequent regimens.	Some patients still experience early peak symptoms or late-cycle symptoms.	Reasonable common starting or maintenance schedule when symptoms and safety markers are stable.	[5-8]
Moderately frequent lower-dose dosing	Twice weekly or three times weekly	Lower dose per injection with smoother expected exposure.	May reduce cyclic symptoms, peak-related adverse effects, and timing-sensitive laboratory interpretation.	More injections; more complex; limited comparative outcome trials.	Consider when weekly dosing is unstable or poorly tolerated, or when symptoms track the injection cycle.	[5-8,31-33]
Highly frequent microdosing	Every other day or daily	Smallest dose per injection and least theoretical fluctuation.	May suit highly fluctuation-sensitive patients or those preferring very small subcutaneous doses.	Greater treatment burden; adherence concerns; limited outcomes data.	Selected patients only. Not necessary when simpler schedules work.	[5-8,31-33]
Long-acting testosterone undecanoate	Usually, every 10-12 weeks after loading	Long depot exposure with a different peak-trough pattern.	Infrequent administration; avoids frequent self-injection.	Slow reversibility; specific logistics and monitoring considerations.	Separate option, not directly equivalent to splitting cypionate or enanthate.	[5,6]

This point should be made cautiously. It does not prove that more frequent injections improve hard outcomes. It simply reminds clinicians that formulation and pharmacokinetics matter when applying safety data to practice. Much of the current work relies on the application of pharmacokinetics rather than clinical trial data.

A practical primary care approach

First, confirm the diagnosis: symptoms, repeated low morning testosterone, total and free or calculated free testosterone where indicated, SHBG, LH, FSH, and an appropriate search for reversible or secondary causes. Establish baseline hematocrit, blood pressure, lipids, fertility goals, and prostate risk where relevant. Seek specialist consultation if necessary to clarify and confirm the diagnosis before starting treatment.

Second, avoid every-two-week dosing as a reflex. If used, it should be reassessed for symptom cycling, peak effects, trough symptoms, and lab timing problems. Third, recognize that weekly dosing is a possible common starting or maintenance schedule for some patients. But be prepared to advise more frequent injections if the patient is willing to accept it.

Fourth, consider more frequent lower-dose regimens when logistically possible and also when it aligns with patient preference. For legacy patients on less frequent injections, consider assessing for signs that indicate more frequent dosing may be helpful: late-cycle symptom recurrence, early-cycle adverse effects, estradiol-related symptoms before adding an aromatase inhibitor, rising hematocrit with high measured levels, blood pressure or fluid retention changes, and low SHBG with discordant symptoms and levels.

Fifth, redistribute before escalating. A low trough should not automatically lead to a higher total weekly dose. The same or a lower weekly amount, given more frequently, may address the problem while reducing peak exposure. After any change, reassess symptoms and repeat laboratory testing with timing clearly documented.

Pitfalls and limitations

Common pitfalls include diagnosing hypogonadism from one low testosterone level, ignoring SHBG, prescribing every-two-week injections by habit, increasing dose because of a trough without asking about peaks, interpreting testosterone without injection timing, treating estradiol numbers rather than symptoms, relying on repeated phlebotomy without reassessing the regimen, and forgetting fertility counseling.

The evidence base also has limits. Few trials directly compare every-two-week, weekly, twice-weekly, every-other-day, and daily dosing for patient-reported outcomes. Data on injection frequency and hematocrit, blood pressure, estradiol, HDL, and cardiovascular outcomes remain limited. Mechanistic plausibility should guide careful practice, not be overstated. Current evidence supports concern about avoidable fluctuation, but it does not support one universal injection schedule.

## Conclusions

Every-two-week injectable testosterone is convenient but pharmacokinetically vulnerable. Weekly dosing can be effective and appropriate, but it should not be considered the only alternative when symptoms, laboratory patterns, or safety markers suggest ongoing fluctuation. More frequent lower-dose dosing is a flexible strategy for willing patients that could potentially mitigate side effect risk. Exogenous testosterone affects erythropoiesis, estradiol, blood pressure, fluid balance, lipids, fertility, and androgen-sensitive tissues. Injectable therapy should therefore be individualized not to maximize testosterone levels but to achieve stable physiologic replacement with the least burden and the safest clinical profile. Future clinical work should seek to further investigate whether higher injection frequency is helpful, using comparative data points or a prospective study.
